# plotXVG: Batch Generation of Publication-Quality Graphs from GROMACS Output

**DOI:** 10.1021/acs.jcim.5c02998

**Published:** 2026-03-10

**Authors:** Måns K. Rosenbaum, David van der Spoel

**Affiliations:** Department of Cell and Molecular Biology, 8097 Uppsala University, Uppsala SE-75124, Sweden

## Abstract

Molecular simulation tools, such as GROMACS, are used routinely to produce time series of energies and other observables. To turn these data into publication-quality figures, a user can either use a (commercial) software package with a graphical user interface, often offering fine control and high-quality output, or write their own code to make plots using a scripting language. In the age of big data and machine learning, it is often necessary to generate many graphs, be able to rapidly inspect them, and make plots for manuscripts. Here, we provide a simple Python tool, plotXVG, built on the well-known Matplotlib plotting library, that will generate publication-quality graphics for line graphs as well as heatmaps and contour plots. This will allow users to rapidly and reproducibly generate a series of graphics files without programming, but a simple application programming interface is available as well for incorporation in, e.g., machine learning applications. Obviously, the tool is applicable to any kind of line graph data or heatmap, not just that from molecular simulations. plotXVG is available as free and open source, which implies that users can extend the tool to their own needs.

## Graphical abstract

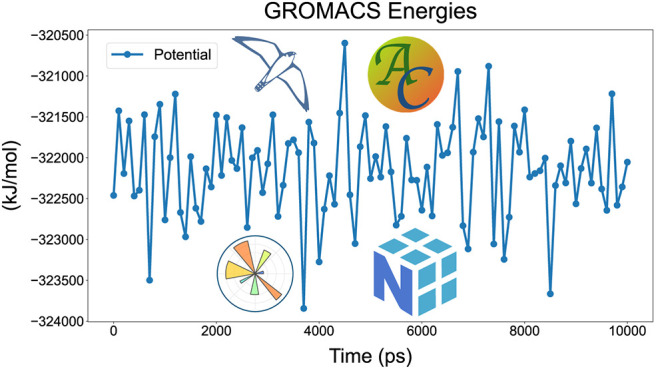

## Introduction

Development of the GROMACS software suite for molecular simulation commenced in the 1990s.^1,2^ At that time, the generation of plots using computer software became accessible through PostScript printers, and to date, GROMACS contains code to directly write encapsulated PostScript files, although it likely is not used a lot in practice. The tool of choice for generating plots on Unix/Linux platforms rapidly became the Grace software.^3^ Grace is a powerful tool, but unfortunately, development of the software has stagnated, and it is difficult, if not impossible, to install it on computers running MacOS or Windows, although it still works on Linux. It should be noted that there have been efforts to make a platform-independent version of Grace, but that may have stagnated as well.^4^ GNUplot is another software with features similar to Grace and, due to continued development, even more powerful.^5^ In addition, there are plotting programs from commercial actors based on graphical interfaces that allow for fine-grained control over individual plots.

In times of big data and machine learning, the demand for reproducibility and accessibility in research has increased significantly, with initiatives, such as FAIR, sharing of data and results from molecular simulation^6^ underscoring this need. An essential part of data analysis is the ability to represent data in an easily interpretable manner. This demands tools that can reproducibly visualize data in a high-quality manner.

We have, therefore, implemented a lightweight script that rapidly generates publication-quality plots from xvg files. With plotXVG, we hope to at least in part replace the Grace software for users of the GROMACS software,^1,2,7,8^ as well as for other simple 1D or 2D data. The software presented here offers more fine-grained control over the output than GMXvg,^9^ and, therefore, is more flexible despite, perhaps, a somewhat higher learning curve. Apart from a command-line interface (CLI), the plotXVG software can be used from other Python scripts using a simple application programming interface (API), as described below.

## Methods

The code consists of about 1000 lines of Python, and it relies on the Matplotlib library^10^ to produce the graphical results, along with NumPy^11^ for some computations and handling of arrays. Usage entails a simple command-line operation:
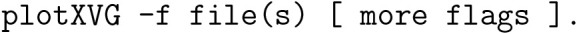


There are many flags available for tailoring a plot to one’s preference, some being to set the minimum and maximum for the *x* and *y* axes, make the *y*-axis logarithmic, write user-defined legends and titles, create histograms, or convert the data to a residual plot by subtracting the *x*-axis. In addition, font sizes for tick markers, axis labels, legends, and the title of the plot can be set. The placement of the labels corresponding to the data can be specified as well. It should be noted that all flags require lowercase letters for correct parsing.

Before plotting, xvg files must be read. In doing this, a small but important subset of the metadata from Grace will be interpreted, including the title of graphs, axes legends, and data set labels, denoted in a short section starting with an ampersand (@). The metadata supported covers most of what is produced by the GROMACS analysis tools. Data sets in one file need to be in columns, starting with the *x* values as column zero, then followed by *y* values for each data set, but can also come after one another, separated by a &. This can look like:
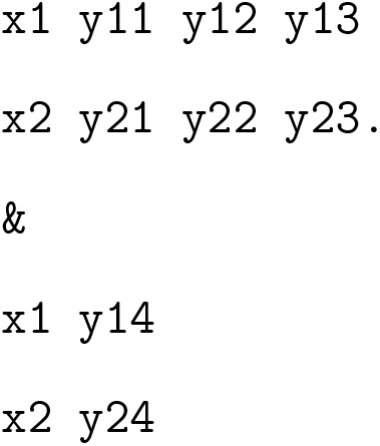


plotXVG will then iterate over input files and format plots after user-specified flags or default arguments are created by employing Matplotlib operations. For further insight into the content of each file, the -debug flag is practical to print file and data set specifics on the terminal output in an easy-to-understand manner. This way, one can keep track of the data sets involved and the actions performed by plotXVG.

Once data are read from one or more files, the plot will appear on the screen of the user, allowing the user to scroll and zoom in interactively and save the plot as a file through the Matplotlib buttons. To allow batch generation of plots, a pdf or png file can be generated at once without showing a window on screen:
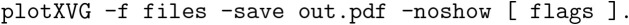


In addition to command-line interaction, the code can be used in the library mode through a simple API.
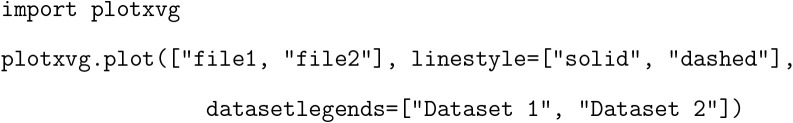


This makes plotting from a script more efficient than repeated ossystem calls from Python. The API may also be useful in Jupyter notebooks or Jupyterlab applications. A plotXVG Manual at GitHub provides more documentation and usage examples.

The OpenMM software, another open-source molecular simulation package,^12,13^ produces energies in csv (comma-separated value) files, which are supported by plotXVG as well. This is true for any file format that stores data using common delimiters, for instance, .dat or .txt. This expands the utility of the program outside the xvg files and can likely be of use for other analysis pipelines.

Adding multiple files as input will automatically produce an all-in-one plot, taking all data sets into consideration. The tool will automatically distinguish all legends by both color and markers or lines. Multiple files can be visualized in panels of plots by adding the –panels flag to the command. This will also produce subplot labels starting from A. Correlation analyses, such as comparing train and test values from machine learning runs, can easily be plotted. By adding the –stats flag for a scatterplot, plotXVG will compute RMSD and *R* ^2^ values using the NumPy library^11^ and add those to the legend. Another feature supported by plotXVG is the ability to create 2D density maps. Using either the –heatmap or –contour flag, plotXVG can compute the probability density *P* using a preferred number of bins and plot it as a heatmap or contour plot. Adding the –gibbs flag, it also calculates Gibbs free energy *G* according to

1 $$G=-k_{B}T\ln P/P_{0}$$

where *P*_0_ is the probability corresponding to the most populated bin, which by definition has *G* = 0, *k*_B_ is Boltzmann’s constant, and *T* is the absolute temperature, which can be specified with a –temperature flag if needed. In this manner, a user can create free energy landscapes from raw scatter data, for instance from a principal component analysis of an MD trajectory.^14,15^ In addition, plotXVG can, by using the Scientific Python (SciPy) library,^16^ estimate the probability density function using kernel density estimation (kde) for data sets with very few data points. These implementations show that plotXVG can be used for broader data analyses for which many users usually rely on other tools. Note that SciPy is not required to use plotXVG except when using the –kde flag.

## Results and Discussion

plotXVG was developed as part of the Alexandria Chemistry Toolkit (ACT^17^) to supplant Grace for plotting simple graphs. As such, it has been used in a number of ACT-related publications already,^18–20^ demonstrating that the results are indeed of publication quality, thanks to the underlying Matplotlib library.^10^

plotXVG reads metadata from an xvg file as input for the plot, which can then be combined with user-specified options. While some learning of the available flags is required, the effort is relatively limited, and information is available from the command line (−help flag) as well as the manual. Most flags are designed for ease-of-use, and all flags have a short and long name, differentiated by using single ″-″ or double ″–″ hyphens. Specific options for utilizing lines and/or markers are specified on the plotXVG command line. Should all data sets be represented as lines, only one line style is needed as input, and plotXVG will generate the rest automatically, along with producing separate colors per data set within panels. This allows for efficient differentiation of various data sets. Plots can be tailored by bestowing each data set with unique arguments for the available flags (Figure 1).

**Figure 1:**
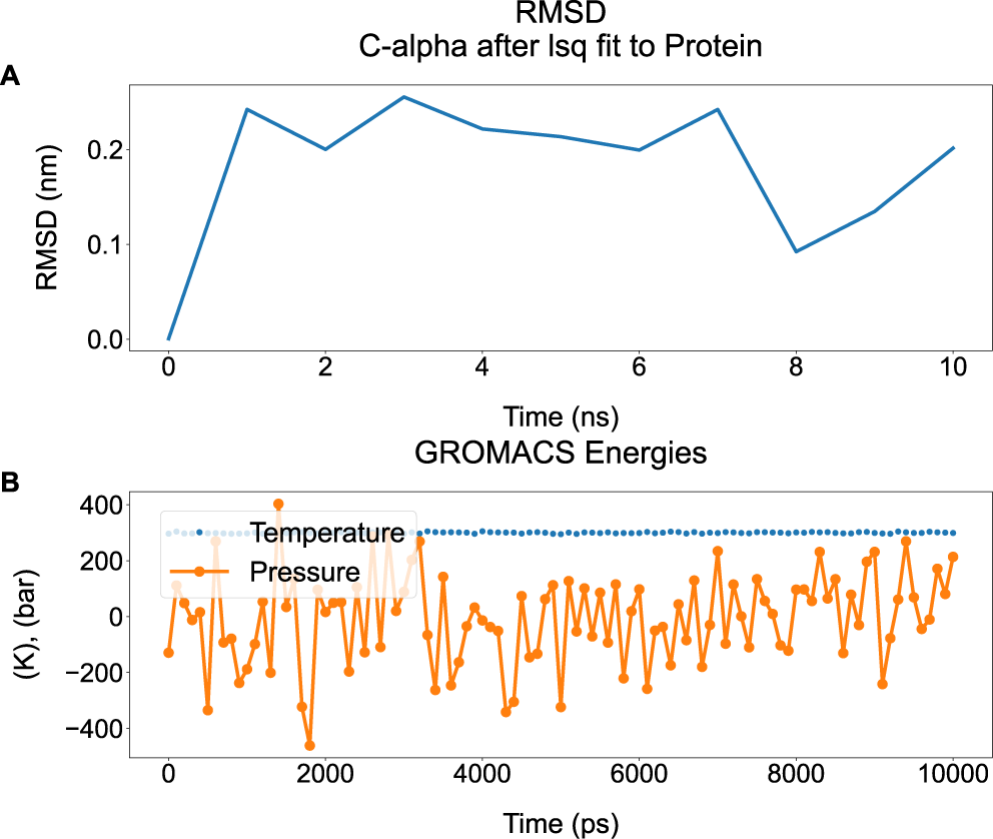
Example graph from a 10 ns simulation of ubiquitin^21^ done using GROMACS.^8^ A) RMSD of C-alpha carbons after least-squares fit to the protein, and B) system temperature and pressure.

In this example, one figure with two subplots was generated. Each panel corresponds to one data file, which, in turn, can contain more than one data set, as apparent in Figure 1B, giving a total of three data sets. Flags governing preferred font and marker sizes, and the size of the figure, can be provided. Figure 1 can be reproduced by executing the following command:
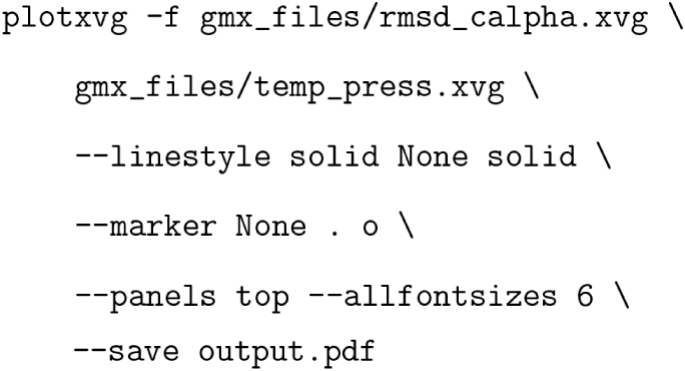


Long flag names are used here for the sake of clarity. Breaking down this command, two files are given as arguments for the –f flag, then some specifics are established. The –linestyle flag parses three arguments: a solid line for data set 1, no line for data set 2 by specifying None, and another solid line for the third data set. Similarly, marker-specific arguments are given by the –marker flag, informing plotXVG that the first data set shall not have a marker, the second one should use small dots, and the third one should use larger dots. Lastly, some extra flags concerning the overall visualization are parsed. The –panels flag says that the two files should be plotted in separate subplots. By default, panels will be plotted on top of each other. The −panels flag can, however, take ″top″ and ″side″ as arguments to create row or column subplots. –allfontsizes simply scales all font sizes by a given number.

Figure 2 shows an example of a correlation plot produced by plotXVG. The data for this correlation plot were generated using the ACT, showing train and test set comparisons of ACT-trained force fields, while the statistical information in the plots was computed by plotXVG. To produce this figure consisting of two subplots, no input flags regarding lines or markers were used, but flags for adding a label to each legend (−data setlegends), for showing statistics in the legend (−stats), and the last one for making the axes equally large (−equalaxes). Figure 2 is reproduced by running:
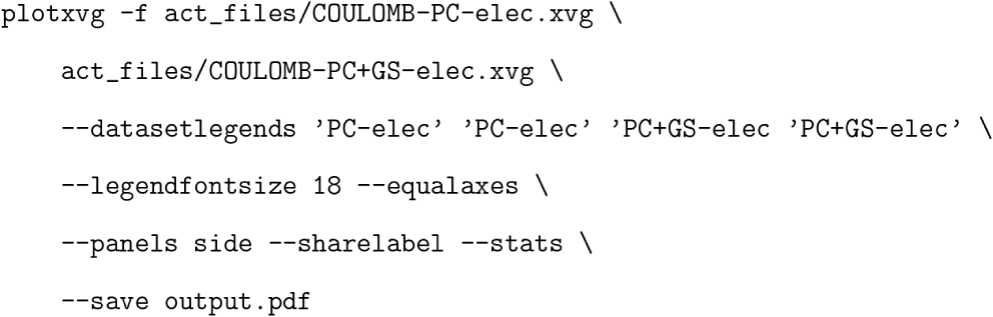


**Figure 2:**
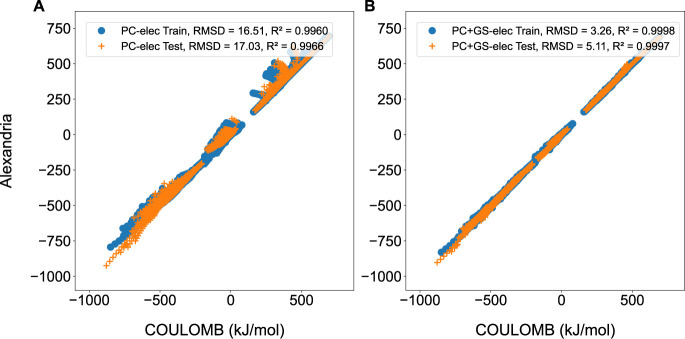
Preliminary results from force field trainings in the ACT using alexandria train_ff, showing Coulomb energies from models generated by the ACT on the *y*-axes and reference energies from symmetry-adapted perturbation theory calculations on the *x*-axes. A) Force field with a point charge model trained on the electrostatic component, and B) force field with a point charge and a Gaussian shell trained on the electrostatic component.

Using the –equalaxes command to make the axes equally large results in square plots, which is useful for correlating data sets. It is worth noting that there is also –squarefig, which, unlike the before mentioned command, makes each plot square by shaping the window itself, adding to the versatility of plotXVG. The –panels flag was, in this example, used with the side argument, leading to the subplots being side by side. In combination with –panels, the –sharelabel flag lets subplots share axis labels. Lastly, the –stats flag will, as mentioned, compute RMSD and *R* ^2^ values from the data.

Contour plots can be created by applying a kernel density estimator (kde) to the data (Figure 4). In this way, a data set with a limited amount of data points can still produce informative results. The commands for generating Figures 3 and 4 using the plotXVG API are
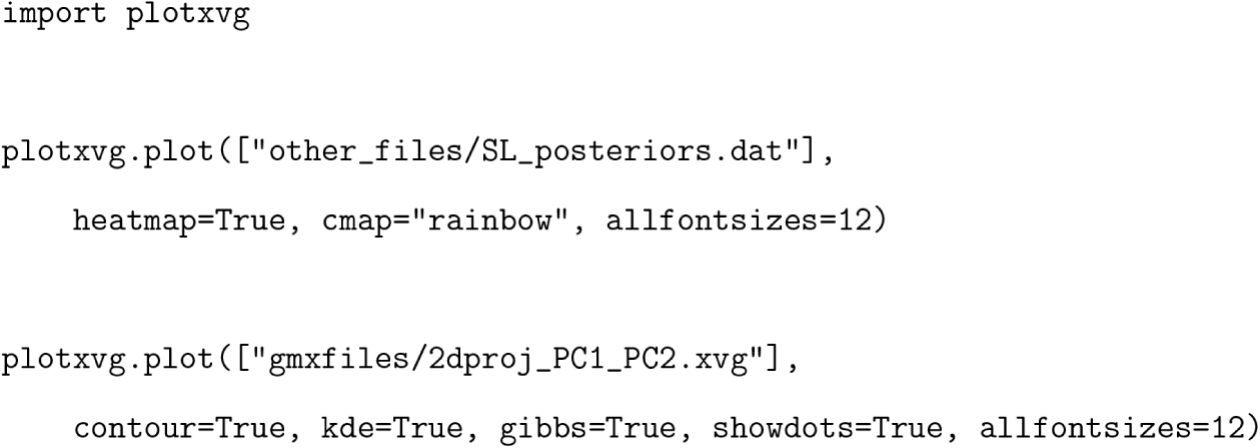


**Figure 3:**
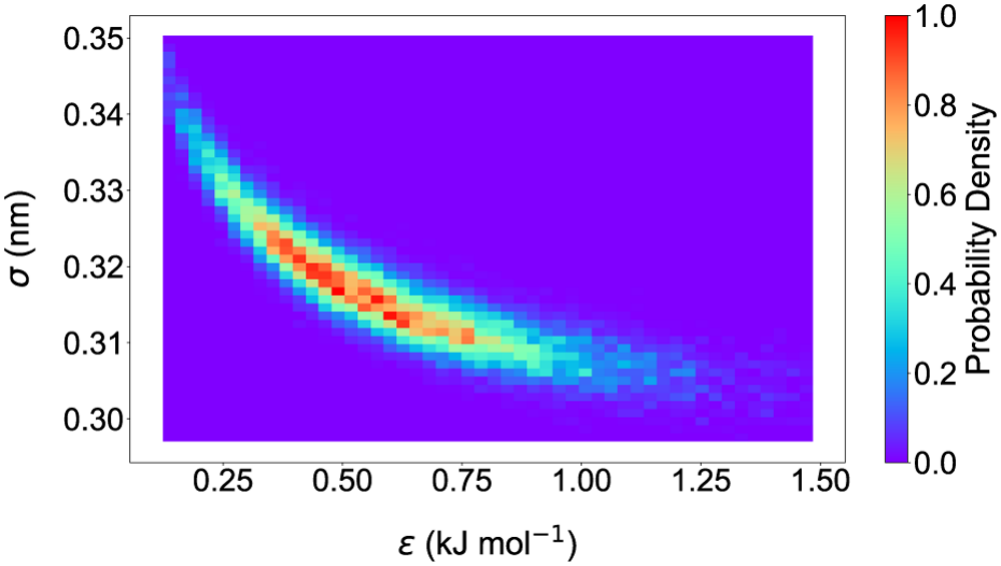
A heatmap created in plotXVG of the synthetic likelihood posteriors from a Bayesian simulation in parameter space. This figure was adapted from an article by Nordman et al.^22^ (Figure 1D).

**Figure 4:**
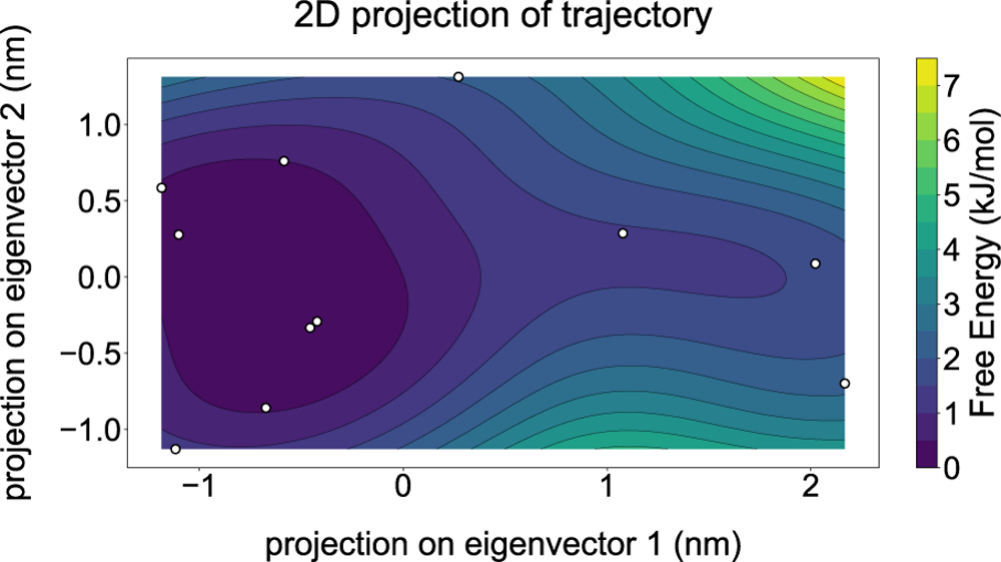
A contour plot, together with the –kde flag, created in plotXVG showing a projection of an MD trajectory of ubiquitin on the two principal components. Simulation and analysis were performed using GROMACS.

Matplotlib supports many colormaps beyond the default gradient colormap viridis used in Figure 4 and rainbow in Figure 3. Available colormaps can be found on their website.^23^ The user can choose any of the above with the –cmap flag. Additional flags for tailoring these 2D density plots are the number of –bins for the heatmap and the number of contour levels using –levels. –showdots adds a scatterplot of the data points on top of the density map. Default values for the number of bins (50), contour levels (15), and temperature (298.15 K) were used to create these plots.

In summary, plotXVG is a simple yet efficient tool to produce high-quality figures using either simple batch commands or a Python script. It incorporates capabilities to create tailored and reproducible figures to match, for instance, the needs of projects related to molecular simulation, while at the same time, it can be used to integrate plotting into data fitting using Scientific Python^16^ or machine learning.^17^

## Data Availability

**Data and Software Availability Statement**: The code and data underlying this study, along with a supplementary plotXVG manual, are freely available on GitHub at https://github.com/AlexandriaChemistry/plotXVG and a permanent copy of release 1.1.1 used for this paper is available from Zenodo.^24^

## References

[1] Bekker, H. ; Berendsen, H. J. C. ; Dijkstra, E. J. ; Achterop, S. ; van Drunen, R. ; van der Spoel, D. ; Sijbers, A. ; Keegstra, H. ; Reitsma, B. ; Renardus, M. K. R. Gromacs: A Parallel Computer for Molecular Dynamics Simulations. In 4th international conference on computational physics (PC 92); World Scientific Publishing: Singapore, 1993, pp. 252–256

[2] Berendsen H. J. C., van der Spoel D., van Drunen R. (1995). GROMACS: A
message-passing parallel molecular dynamics implementation. Comput. Phys. Commun..

[3] Grace User’s Guide The Grace Team; 2008; https://plasma-gate.weizmann.ac.il/Grace/.

[4] Winter, A. Native Grace for Windows, Linux and Mac OS X based on Qt; 2022; https://sourceforge.net/projects/qtgrace/.

[5] Williams, T. ; Kelley, C. gnuplot 6 0.0. In An Interactive Plotting Program; 2024; http://www.gnuplot.info/docs_6.0/Gnuplot_6.pdf.

[6] Amaro R. E. (2025). The need to implement
FAIR principles in biomolecular simulations. Nat. Methods.

[7] van
der Spoel D., Lindahl E., Hess B., Groenhof G., Mark A. E., Berendsen H. J. C. (2005). GROMACS: Fast, Flexible and Free. J. Comput. Chem..

[8] Abraham M. J., Murtola T., Schulz R., Páll S., Smith J. C., Hess B., Lindahl E. (2015). GROMACS: High performance
molecular simulations through multi-level parallelism from laptops
to supercomputers. SoftwareX.

[9] Sahu, V. K. ; Rathore, V. ; Lin, W.-W. ; Ranjan, A. ; Basu, S. GMXvg: A tool to plot GROMACS.xvg files; 2025; https://github.com/TheBiomics/GMXvg.

[10] Hunter J. D. (2007). Matplotlib:
A 2D graphics environment. Comput. Sci. Eng..

[11] Harris C. R. (2020). Array programming with
NumPy. Nature.

[12] Eastman P., Pande V. S. (2010). OpenMM: A Hardware-Independent
Framework for Molecular
Simulations. Comput. Sci. Eng..

[13] Eastman P. (2024). OpenMM 8: Molecular
Dynamics Simulation with Machine Learning Potentials. J. Phys. Chem. B.

[14] Garcia A. E. (1992). Large-Amplitude
Nonlinear Motions in Proteins. Phys. Rev. Lett..

[15] Amadei A., Linssen A. B. M., Berendsen H. J. C. (1993). Essential
Dynamics of Proteins. Proteins:Struct., Funct.,
Bioinf..

[16] Virtanen P. (2020). SciPy 1.0: Fundamental Algorithms for Scientific Computing in Python. Nat. Methods.

[17] van
der Spoel D., Marrades J., Kříž K., Hosseini A. N., Nordman A. T., Martins J. P. A., Walz M.-M., van Maaren P. J., Ghahremanpour M. M. (2025). Evolutionary Machine Learning of
Physics-Based Force Fields in High-Dimensional Parameter-Space. Digital Discovery.

[18] Kříž K., van der Spoel D. (2024). Quantification of Anisotropy in Exchange and Dispersion
Interactions: A Simple Model for Physics-Based Force Fields. J. Phys. Chem. Lett..

[19] van
der Spoel D., Hosseini A. N. (2025). Point + Gaussian charge model for
electrostatic interactions derived by machine learning. Phys. Chem. Chem. Phys..

[20] van
Maaren P. J., van der Spoel D. (2025). Quantitative Evaluation of Anharmonic
Bond Potentials for Molecular Simulations. Digital
Discovery.

[21] Ramage R., Green J., Muir T. W., Ogunjobi O. M., Love S., Shaw K. (1994). Synthetic, structural
and biological studies of the ubiquitin system:
The total chemical synthesis of ubiquitin. Biochem.
J..

[22] Nordman A.
T., Engblom S., van der Spoel D. (2025). Bayesian Three Point Water. Npj
Comput. Mater..

[23] Matplotlib Choosing Colormaps in Matplotlib; https://matplotlib.org/stable/users/explain/colors/colormaps.html.

[24] Rosenbaum, M. ; van der Spoel, D. plotXVG: Batch-generation of publication quality graphs from GROMACS output; plot XVG 1.1.1, zenodo, 2026. 10.1021/acs.jcim.5c02998 PMC13014445 41804073

